# Antibiotic susceptibility and molecular epidemiology of *Acinetobacter calcoaceticus–baumannii* complex strains isolated from a referral hospital in northern Vietnam

**DOI:** 10.1016/j.jgar.2014.05.003

**Published:** 2014-12

**Authors:** Trang Dinh Van, Quynh-Dao Dinh, Phu Dinh Vu, Trung Vu Nguyen, Ca Van Pham, Trinh Tuyet Dao, Cam Dac Phung, Ha Thu Thi Hoang, Nga Thi Tang, Nga Thuy Do, Kinh Van Nguyen, Heiman Wertheim

**Affiliations:** a National Hospital of Tropical Diseases, Hanoi, Viet Nam; b Oxford University Clinical Research Unit, Wellcome Trust Major Overseas Program, Hanoi, Viet Nam; c National Institute of Hygiene and Epidemiology, Hanoi, Viet Nam; d Nuffield Department of Clinical Medicine, Centre for Tropical Medicine, University of Oxford, Oxford, UK

**Keywords:** *Acinetobacter baumannii*, Ventilator-associated pneumonia, Hospital-acquired infection, Antibiotic resistance, Genotype

## Abstract

*Acinetobacter calcoaceticus–baumannii* complex is a common cause of hospital-acquired infections (HAIs) globally, remarkable for its high rate of antibiotic resistance, including to carbapenems. There are few data on the resistance of *A. baumannii* in Vietnam, which are essential for developing evidence-based treatment guidelines for HAIs. Antibiotic susceptibility testing was conducted by VITEK^®^2, and pulsed-field gel electrophoresis (PFGE) was performed on 66 clinical *A. baumannii* complex isolates recovered during 2009 at the National Hospital of Tropical Diseases (NHTD), a referral hospital in Hanoi, Vietnam. Basic demographic and clinical data were collected and analysed using descriptive statistics. Most isolates came from lower respiratory tract specimens (59; 89.4%) from intensive care unit (ICU) patients [64/65 (98.5%) with available data] who had been admitted to NHTD for ≥2 days [42/46 (91.3%) with available data]. More than 90% of the isolates were resistant to the tested β-lactamase/β-lactamase inhibitors, cephalosporins, carbapenems, fluoroquinolones and trimethoprim/sulfamethoxazole. Moreover, 25.4% (16/63) were resistant to all tested β-lactams, quinolones and aminoglycosides. All isolates remained sensitive to colistin and 58.7% were susceptible to tigecycline. Of the 66 isolates, 49 could be classified into eight PFGE types (A–H). Every PFGE type, except D, had cluster(s) of three or more isolates with a temporal relationship. In conclusion, these data suggest a significant rise in *A. baumannii* antibiotic resistance in Vietnam. Clustering within PFGE types supports cross-transmission of *A. baumannii* within the ICU at NHTD. Increased research and resources in optimising treatment, infection control and antibiotic stewardship are needed.

## 1 Introduction

*Acinetobacter calcoaceticus–baumannii* complex is emerging as one of the most common causes of hospital-acquired infections (HAIs) in intensive care units (ICUs) worldwide and is often resistant to multiple antibiotic classes, complicating treatment [1]. *Acinetobacter baumannii* is a strictly aerobic, non-motile, Gram-negative bacillus belonging to the *A. calcoaceticus–baumannii* complex within the family Moraxellaceae of the order Gammaproteobacteria. Identification by phenotypic methods or DNA–DNA hybridisation does not reliably distinguish *A. baumannii* from other members of the *A. calcoaceticus–baumannii* complex (henceforth referred to as *A. baumannii*) [1]. This poses challenges clinically because *A. baumannii* can be pathogenic whereas *A. calcoaceticus* is environmental. The exact reservoir of *A. baumannii* remains undefined [2]. In contrast to other *Acinetobacter* spp., *A. baumannii* is uncommon in nature compared with the hospital environment [1]. *A. baumannii* is able to survive on dry surfaces in hospital environments for up to 4 months [3].

*A. baumannii* infection occurs when the immunological barriers of the host are breached (e.g. mechanical ventilation) and is hence considered an opportunistic pathogen [4]. *A. baumannii* causes various types of HAI, including ventilator-associated pneumonia (VAP), bacteraemia, urinary tract infection, meningitis, and infections complicating burn wounds [5]. Treatment is difficult due to the high rates of antibiotic resistance worldwide, including resistance to carbapenems [2]. However, few data are available on the molecular epidemiology and antibiotic resistance of *A. baumannii* infections in Asia, including Vietnam. Rapid development in Vietnam has been accompanied by the increasing availability of complex healthcare, including ICUs, and accompanied by HAIs. As the isolation of *A. baumannii* is reported to be particularly common in VAP, it is crucial to know more regarding the resistance and epidemiology of these bacteria in a resource-constrained setting such as Vietnam. This study describes the antibiotic susceptibility and molecular epidemiology of *A. baumannii* isolates from a referral hospital in Hanoi, Vietnam.

## 2 Materials and methods

### 2.1 Design

This was a retrospective study examining the molecular epidemiology and antibiotic susceptibility of clinical *A. baumannii* isolates collected from hospitalised patients from January to December 2009 at the National Hospital for Tropical Diseases (NHTD) in Hanoi. The NHTD is a tertiary referral hospital for adult infectious diseases with a catchment area that covers Hanoi and the surrounding northern provinces. At the time of the study, NHTD had 150 inpatient beds including 15 ICU beds, and ca. 4000 inpatient admissions annually. The study was approved by the Scientific and Ethical Committee of NHTD.

### 2.2 Isolates

In this study, 66 stored *A. baumannii* isolates cultured from unique patients admitted to NHTD in 2009 were viable and available for study, from a total of 99 *A. baumannii* isolates. For each patient, the first isolate was selected and isolates from sterile sites were chosen over respiratory samples, whenever possible. *A. baumannii* isolates were identified by standard microbiological methods, including API 20 NE (bioMérieux, Marcy-l’Étoile, France). PCR targeting the *bla*_OXA-51-like_ gene was conducted later and was positive in 48 of the 48 available isolates [6]. Isolates were stored in glycerol medium at −70 °C. In total, 63 isolates were subjected to antibiotic susceptibility testing according to Clinical and Laboratory Standards Institute (CLSI) guidelines using the VITEK^®^2 system with card AST-N089 22237 (bioMérieux) for the following antibiotics: ampicillin; amoxicillin/clavulanic acid; piperacillin/tazobactam; ceftazidime; cefotaxime; cefepime; imipenem; meropenem; amikacin; gentamicin; tobramycin; ciprofloxacin; levofloxacin; trimethoprim/sulfamethoxazole (SXT); colistin; and tigecycline. This antibiotic card interprets tigecycline susceptibility according to US Food and Drug Administration (FDA) breakpoints for Enterobacteriaceae. In total, 58 isolates were typed by pulsed-field gel electrophoresis (PFGE) as previously described using the restriction enzyme *Apa*I [7], [8]. In addition, data were collected on specimen source and date of collection. From the patient chart, data were collected on patient demographics, location, and brief clinical data such as type of infection.

### 2.3 Analysis

Susceptibility data were analysed using WHOnet 5.5 software (World Health Organization, Geneva, Switzerland). Amikacin data are not shown as these are considered to be unreliable when tested by the VITEK system [9]. Analysis of PFGE results was done visually using the criteria of Tenover et al. [10] by two readers (VDT and HW) and discrepancies were resolved by a third reader (QD). Isolates with PFGE patterns that were indistinguishable (zero genetic differences) or closely related (one genetic difference) were grouped together. Isolate and patient data were analysed using Microsoft Excel 2010 (Microsoft Corp., Redmond, WA) using descriptive statistics as appropriate.

## 3 Results

### 3.1 Source of the *A. baumannii* isolates

The 66 *A. baumannii* isolates came from the lower respiratory tract (59; 89.4%), blood (6; 9.1%) and pus (1; 1.5%). The median patient age was 51 years (range 17–93 years) and the majority of patients were male (48; 72.7%). The 59 *A. baumannii* isolates from sputum comprised 13.1% of a total of 451 sputum specimens. Almost all patients were admitted to the ICU (64/65 with available data). The reason(s) for ICU admission was available for 43 patients (some patients had more than one reason for ICU admission listed) and included pneumonia in 14 (32.6%), sepsis or septic shock in 10 (23.3%), tetanus in 9 (20.9%), central nervous system infection in 8 (18.6%), and substance abuse, respiratory failure not otherwise specified, dengue fever, and fever not otherwise specified each in 2 patients (4.7%). Most specimens were collected for clinical suspicion of VAP. Most specimens were collected ≥2 days after the patient's admission to NHTD [42/46 (91.3%) where dates of admission and specimen collection were both available] and were therefore compatible with HAI. Patient information for three of the remaining specimens indicated that the patients had been transferred from another hospital; however, the length of stay at the referring hospital was not available.

### 3.2 Antibiotic susceptibility

In total, 63 isolates underwent antibiotic susceptibility testing using the VITEK^®^2 system (Table 1). Resistance rates were high, with >90% of isolates being resistant to the tested β-lactamase/β-lactamase inhibitors, cephalosporins, carbapenems, fluoroquinolones and SXT. Moreover, 34.9% of isolates were resistant to both gentamicin and tobramycin, and 25.4% of isolates (*n* = 16) were resistant to all tested β-lactams, quinolones and aminoglycosides. All isolates remained sensitive to colistin and 58.7% were susceptible to tigecycline.

**Table 1:** Antibiotic susceptibility testing of 63 *Acinetobacter baumannii* isolates at the National Hospital of Tropical Diseases (Hanoi, Vietnam), 2009.

Antibiotic	Susceptible		Intermediate		Resistant	
	%	*n*	%	*n*	%	*n*
AMC	0.0	0	0.0	0	100.0	63
TZP	1.6	1	3.2	2	95.2	60
Cefotaxime	0.0	0	1.6	1	98.4	62
Ceftazidime	0.0	0	1.6	1	98.4	62
Cefepime	0.0	0	1.6	1	98.4	62
Imipenem	7.9	5	0.0	0	92.1	58
Meropenem	7.9	5	0.0	0	92.1	58
Gentamicin	17.5	11	27.0	17	55.6	35
Tobramycin	31.7	20	25.4	16	42.9	27
Ciprofloxacin	1.6	1	0.0	0	98.4	62
Levofloxacin	1.6	1	7.9	5	90.5	57
Tigecycline	58.7	37	38.1	24	3.2	2
Colistin	100.0	63	0.0	0	0.0	0
SXT	0.0	0	0.0	0	100.0	63

AMC, amoxicillin/clavulanic acid; TZP, piperacillin/tazobactam; SXT, trimethoprim/sulfamethoxazole.

### 3.3 Pulsed-field gel electrophoresis

Of the 58 isolates that underwent PFGE, 49 were classified into eight PFGE types and were assigned the letters A–H (Supplementary Fig. S1). The letter assignment is not intended to ascribe relatedness among the PFGE types. PFGE type A isolates (*n* = 6) originated from sputum, type B (*n* = 7) from sputum (4) and blood (3), type C (*n* = 15) from sputum (14) and blood (1), and types D (*n* = 3), E (*n* = 4), F (*n* = 4), G (*n* = 5) and H (*n* = 5) were all from sputum. The remaining nine isolates, including two blood isolates, did not appear related to other isolates by PFGE.

Supplementary Fig. I related to this article can be found, in the online version, at doi:10.1016/j.jgar.2014.05.003.

**Supplementary Fig. I:**
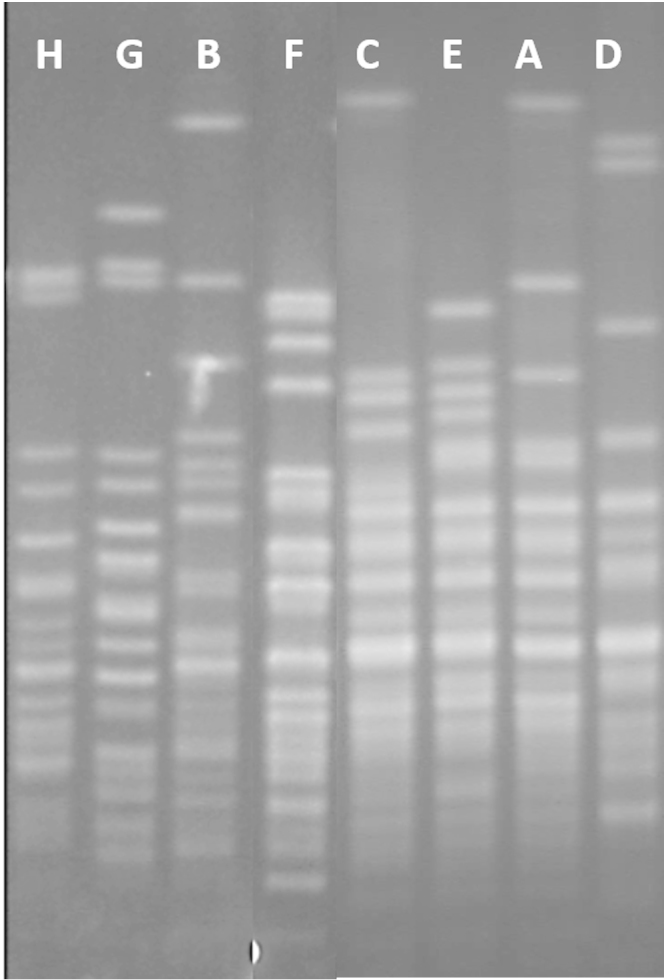
Representative pulsed-field gel electrophoresis (PFGE) patterns of each *Acinetobacter baumannii* type (A–H), digested with *Apa*I. Isolates with PFGE patterns that were indistinguishable (zero genetic differences) or closely related (one genetic difference) were grouped into a type. Letter ordering is not intended to ascribe relatedness of types.

**Fig. 1:**
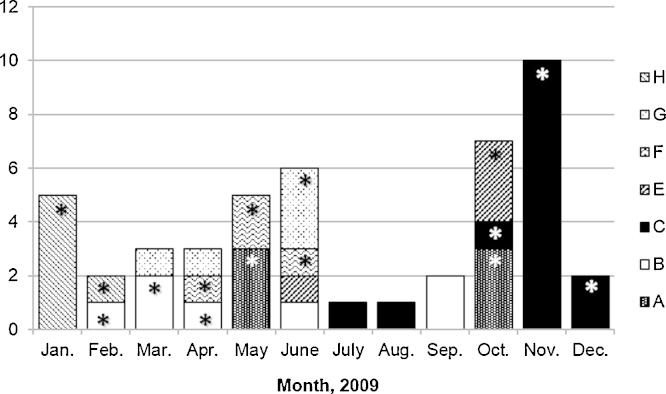
Clustering of *Acinetobacter calcoaceticus–baumannii* complex isolates in 2009 at the National Hospital of Tropical Diseases (Hanoi, Vietnam) within pulsed-field gel electrophoresis (PFGE) types A–C and E–H. * Indicates cluster isolates.

Every PFGE type, except D type, had cluster(s) of three or more isolates that were temporally related, with time spans ranging from 6 days to 67 days (Fig. 1). On average, isolates within each cluster were detected every 2–16.8 days. Type A isolates, which clustered in May and October, had identical antibiotic testing results, with susceptibility only to tigecycline and colistin (Table 2). Isolates within the other PFGE types differed in antibiotic resistance by at least one antibiotic class, most often the aminoglycosides, but also the carbapenems, quinolones and tigecycline. The type B and C clusters included all the blood isolates from those types.

**Table 2:** Antibiotic susceptibility patterns^a^ according to pulsed-field gel electrophoresis (PFGE) type.

Antibiotic/antibiotic class	PFGE type							
	A (6)	B^b^ (7)	C (15)	D (3)	E (4)	F (4)	G^b^ (5)	H (5)
β-Lactamase/β-lactamase inhibitors	R	R	R	R	R	R	R	R
Cephalosporins	R	R	R	R	R	R	R	R
Carbapenems	R	r/s	R	R	R	R	R	R
Gentamicin	R	r/i/s	r/i	R	R	R	r/i/s	r/i
Tobramycin	R	r/s	r/i/s	i/s	R	r/i	r/s	i/s
Fluoroquinolones	R	R	R	R	r/i	R	R	R
Tigecycline	S	S	I	i/s	i/s	S	S	r/i/s
Colistin	S	S	S	S	S	S	S	S
SXT	R	R	R	R	R	R	R	R

R, resistant; I, intermediate; S, susceptible; SXT, trimethoprim/sulfamethoxazole.

a Susceptibility phenotypes reported in non-capitalised letters and separated by a slash indicate varying susceptibility patterns within that cluster of strains.

b Antibiotic testing data were missing for one isolate.

## 4 Discussion and conclusion

This study presents antibiotic susceptibility data for 63 *A. baumannii* isolates at a referral hospital in Hanoi, Vietnam, in 2009. The majority of isolates came from sputum samples collected from ICU patients ≥2 days into their admission at NHTD. There were high rates of resistance, including nearly complete resistance to all tested β-lactam antibiotics. Susceptibility to colistin and tigecycline remained high. The VITEK 2 system used in this study is considered reliable for most antibiotics, including colistin [11]. Amikacin was excluded from this analysis as testing for amikacin susceptibility by VITEK 2 was previously shown to produce very major errors more than one-third of the time compared with broth microdilution [9].

Data from northern Vietnam on *A. baumannii* isolates from 1997 to 1998 showed considerably less resistance, with resistance rates of ca. 50% for cephalosporins, 50% for aminoglycosides, 25% for quinolones and 50% for SXT (Dr. Đoàn Mai Phương, Bach Mai Hospital, Vietnam, unpublished data). These data suggest a sharp increase in *A. baumannii* resistance over the last 10 years in Vietnam. This is supported by the COMPACT II study, which reported 89.5% carbapenem-non-susceptibility by Etest among 19 *A. baumannii* isolates from Vietnam, comparable with the current results [12]. In a recent study at a hospital in western China, 31 *A. baumannii* isolates from the ICU were also highly resistant, including 96.3% to SXT, 92.6% to cefotaxime and 55.6% to carbapenems [13].

These results raise questions regarding what constitutes appropriate empirical and definitive treatment of highly resistant *A. baumannii* infections, including the use of combination therapy and colistin. In this study, rifampicin was not tested, which is active alone and in combination in in vitro and animal studies [14], [15]. However, clinical data on the use of rifampicin in combination has generally been non-comparative. At NHTD, testing amikacin susceptibility using a more reliable method may broaden the available therapeutic options. Meanwhile, there is increasing reliance on colistin, even though it is not always readily available in Vietnam and is relatively costly. A global colistin usage survey indicated that in Vietnam, no loading dose of colistin is given, which may lead to treatment failure [16]. In general, underdosing of colistin risks the development of bacterial resistance against this last-line drug (unpublished data).

Numerous PFGE types were identified, suggesting a diverse population of *A. baumannii* rather than the spread of a specific clone. Nevertheless, PFGE types C and H appeared dominant, clustering from October to December and January to February, respectively. Confidence in the smaller clusters was limited by the sample size. The clonality of these PFGE types is supported by similarities in antibiotic susceptibility profiles. The clusters suggest transmission within the ICU with varying *A. baumannii* types in 2009 and emphasise the importance of rigorous infection control.

This study has limitations that need to be acknowledged. Limitations in microbiological and clinical data could have led to the inclusion of isolates from specimens that represented colonisation and contamination rather than infection. We also did not have details on where patients were located within the ICU to prove environmental, healthcare worker, or direct patient-to-patient spread of infection. Furthermore, we did not distinguish between pathogenic *A. baumannii* species from the environmental species *A. calcoaceticus.* A significant number of isolates were either not viable or were not groupable by PFGE, limiting the detection of PFGE types and clusters. However, we do not expect any selection bias in the isolates tested, which do provide us important information on the resistance patterns of *A. calcoaceticus–baumannii* complex strains in Vietnam.

These data show that *A. calcoaceticus*–*baumannii* complex is an important cause of HAIs in ICUs in northern Vietnam. The high rates of antibiotic resistance present a major opportunity for conducting treatment trials to determine the optimal treatment of *A. baumannii* HAIs, including the best timing and dosing of colistin as well as the role of combination therapy. Given the temporal clustering of related *A. baumannii* types, adequate resources need to be dedicated towards antibiotic stewardship and infection control activities. We are undertaking further studies on VAP and a nationwide HAI surveillance programme, which will inform future steps.

## Funding

This research project was made possible with funds from the Global Antibiotic Resistance Partnership (USA) and the 10.13039/100004440Wellcome Trust (UK).

## Competing interests

None declared.

## Ethical approval

This study was approved by the Scientific and Ethical Committee of the National Hospital for Tropical Diseases (Hanoi, Vietnam).
